# Pruning and Tending Immune Memories: Spacer Dynamics in the CRISPR Array

**DOI:** 10.3389/fmicb.2021.664299

**Published:** 2021-04-01

**Authors:** Sandra C. Garrett

**Affiliations:** Department of Genetics and Genome Sciences, Institute for Systems Genomics, UConn Health, Farmington, CT, United States

**Keywords:** adaptation, spacer acquisition, repeat, array, CRISPR, spacer deletion

## Abstract

CRISPR-Cas (Clustered Regularly Interspaced Short Palindromic Repeats and CRISPR-associated genes) is a type of prokaryotic immune system that is unique in its ability to provide sequence-specific adaptive protection, which can be updated in response to new threats. CRISPR-Cas does this by storing fragments of DNA from invading genetic elements in an array interspersed with short repeats. The CRISPR array can be continuously updated through integration of new DNA fragments (termed spacers) at one end, but over time existing spacers become obsolete. To optimize immunity, spacer uptake, residency, and loss must be regulated. This mini-review summarizes what is known about how spacers are organized, maintained, and lost from CRISPR arrays.

## Introduction

Prokaryotes have evolved a diverse repertoire of tools to restrict the proliferation of deleterious mobile genetic elements (Koonin et al., 2017). Uniquely among these tools, CRISPR-Cas (Clustered Regularly Interspaced Short Palindromic Repeats and CRISPR-associated genes) provides sequence-specific protection that can be updated in the face of novel threats, making it an adaptive immune system. CRISPR stores sequence information about potentially parasitic or harmful mobile genetic elements in an array (Barrangou et al., 2007) and uses that information to carry out targeted degradation of DNA or RNA, depending upon CRISPR type (Makarova et al., 2020). CRISPR-Cas systems are diverse and have been classified into two classes, six distinct types (I–VI), and at least 33 subtypes (Makarova et al., 2020), but certain characteristics are shared. All CRISPR arrays contain a series of direct repeats separated by short sequences called “spacers” which match DNA from previously encountered invaders (Bolotin et al., 2005; Mojica et al., 2005; Pourcel et al., 2005). An upstream leader sequence regulates transcription of the array and mediates addition of new spacers (Jansen et al., 2002; Yosef et al., 2012; Wei et al., 2015; Alkhnbashi et al., 2016). In addition to the CRISPR array, there are usually nearby genes encoding CRISPR-associated (Cas) proteins, including nucleases.

After transcription, CRISPR array RNAs are processed into short guide RNAs (crRNAs) which associate with Cas nucleases to form a crRNA-guided effector complex (Hille et al., 2018). The crRNA base pairs with its complementary sequence in the target DNA or RNA (termed the “protospacer” since it corresponds to the invader nucleic acid that was originally captured and stored as a spacer) and leads to its degradation (interference). For DNA-targeting CRISPR systems, there must be a short activating sequence next to the target (called the Protospacer Adjacent Motif or PAM) for efficient interference (Deveau et al., 2008; Mojica et al., 2009; Shah et al., 2013). New spacers are added to the array in a process called adaptation, wherein two proteins, Cas1 and Cas2, integrate fragments of DNA (McGinn and Marraffini, 2019) to produce new immune memories.

While the field has made great gains in understanding interference and adaptation in a wide range of organisms, many questions remain. For one, how are the individual immune memories in this heritable and adaptable system maintained over time? New spacers are continuously added in response to novel threats, but most arrays are less than 30 spacers long, suggesting that some immune memories are purged—which ones and how? This review will examine what we have learned about the dynamics of CRISPR arrays, with a focus on how immune memories (the spacers) are organized, maintained, and lost.

### CRISPR Arrays Are Uniquely Organized Sequence Storage Banks

**FIGURE 1:**
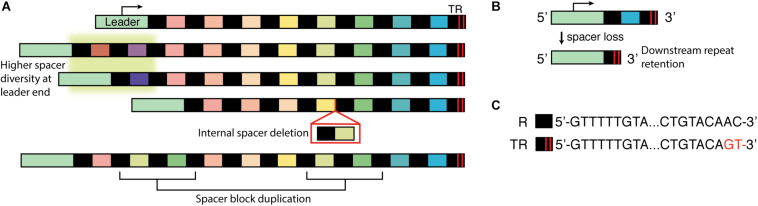
Cartoon diagram of CRISPR array organization and key processes. **(A)** CRISPR arrays from related strains or isolates were compared; differences in spacer composition illustrated three key characteristics: highest spacer diversity is found at the leader-adjacent end of the array (highlighted in *green*), missing spacers in the array suggest spacer deletion events, and repeated blocks or repeated individual spacers suggests duplication events. **(B)** Sequencing showed that the downstream repeat is maintained after an upstream spacer-repeat unit deletion. **(C)** Example internal repeat (R) and terminal repeat (TR) for a type II-A CRISPR array from *Streptococcus thermophilus*. The 3′ end (leader distal) has two nucleotide substitutions, which disrupt dyad symmetry at the ends.

The most notable component of CRISPR-Cas systems is the repeat-spacer array, and the unusual structure of these elements was the first component of CRISPR-Cas to capture researchers attention as they studied nearby genes in *Escherichia coli* (Ishino et al., 1987; Nakata et al., 1989). Other types of repeats had been described in prokaryotic genomes, but in these new elements they found a novel layout: about a dozen direct repeats with loose dyad symmetry were arranged in a regularly spaced array (Figure 1A). The repeats were identical (or nearly identical) in sequence and length, while the intervening spacers had a common length but seemingly random sequence. The authors searched for and found the repeats in genomes of two other species of gram-negative bacteria and other groups found similar repeats in a range of bacteria and archaea (Groenen et al., 1993; Mojica et al., 1993; Mojica et al., 1995; Masepohl et al., 1996; Hoe et al., 1999). The broad distribution and surprisingly well-conserved layout suggested an important functional role for CRISPR arrays (Mojica et al., 2000; Jansen et al., 2002). That role was uncovered through a key observation about spacers: their sequences often matched DNA of mobile genetic elements like plasmids, phages, and prophages. Thus the CRISPR array appeared to be part of an immune system, with the spacer sequences acting as immune memories (Bolotin et al., 2005; Mojica et al., 2005; Pourcel et al., 2005). This immune function was then confirmed directly. In a lab setting, cultures of a CRISPR-endowed strain of *Streptococcus thermophilus* were almost entirely killed off by lytic phage, but the small number of survivors (bacteriophage-insensitive mutants, BIM) all had at least one new spacer which matched the phage genome (Barrangou et al., 2007).

These and other studies showed that CRISPR-Cas could function as an immune system, and they also began to reveal general characteristics of how new spacers were acquired and stored. First, while studying bacteriophage-insensitive mutants it was noted that new spacers were added to one end of the array (Barrangou et al., 2007); this end contained the “leader,” a 200–300 bp stretch of non-coding DNA (Jansen et al., 2002), which was later shown to regulate spacer uptake and array transcription (Pougach et al., 2010; Yosef et al., 2012). During uptake of a new spacer, the repeat was duplicated, so that an entire spacer-repeat unit was added (Barrangou et al., 2007). Later work showed that new spacer-repeat units could occasionally be added to the interior of the array, termed “ectopic” integration. In rare examples where ectopic integrations appear to outnumber leader-adjacent events, mutations in the leader were found and thought to cause the atypical localization (McGinn and Marraffini, 2016). Recently, ectopic integrations were reported in type II systems of *S. thermophilus* in the absence of leader mutations. While most (83%) integrations were leader-adjacent, the minority of ectopic events show that polarity is typical but not always absolute (Achigar et al., 2021).

Polarity of spacer uptake was not unique to lab–cultured organisms. Whenever CRISPR arrays from related strains were compared, a common pattern emerged: the greatest diversity of spacers was observed near the leader, with many of those spacers being unique to one strain or another, while the distal end of the array tended to have a series of spacers that was shared by many strains (Figure 1A; Pourcel et al., 2005; Lillestol et al., 2006; Horvath et al., 2008; Held et al., 2010; Lopez-Sanchez et al., 2012; Lier et al., 2015; Rao et al., 2016). This pattern supported a polar and sequential process of spacer addition, with recent events near the leader and ancestral events at the distal end. A careful comparison of *Sulfolobus islandicus* isolates from a single hot spring lent particular support for this model: when two arrays shared non-identical spacers that likely arose from the same viral invader, the spacers were often in the same relative position within their respective arrays. The spacers’ positions appeared to serve as a time stamp for the moment when the virus appeared in the spring and was captured into the CRISPR arrays (Held et al., 2010).

Second, it became clear that spacer-repeat units could be duplicated or deleted from the array. These changes were often observed in the middle of the array, while the distal end (termed “trailer” or “anchor” end) was typically conserved (Pourcel et al., 2005; Lillestol et al., 2006; Horvath et al., 2008; Lopez-Sanchez et al., 2012; Weinberger et al., 2012; Lam and Ye, 2019; Deecker and Ensminger, 2020). Evidence of losses or duplications was first inferred by comparing arrays from related strains; arrays that differed only by the absence of one or more contiguous spacers were thought to be the result of deletions (Figure 1A; Pourcel et al., 2005; Lillestol et al., 2006; Held et al., 2010; Gudbergsdottir et al., 2011; Lopez-Sanchez et al., 2012; Achigar et al., 2017). Spacer deletion was also sometimes detected while sequencing bacteriophage survivors (Deveau et al., 2008; Achigar et al., 2017). A minority of survivors both lost a contiguous block of existing spacers and added a new spacer against the experimental phage, leading some authors to suggest that “spacer deletion may occur concomitantly with the addition of new spacers” (Deveau et al., 2008). Repeated blocks of spacers were presumed to be duplications rather than independent adaptation events (Bolotin et al., 2005; Lillestol et al., 2006; Held et al., 2010; Lopez-Sanchez et al., 2012; Lier et al., 2015; Stout et al., 2018). While a second encounter with an old invader could conceivably lead to uptake of the same spacer twice, an identical series of spacers is unlikely.

Spacer deletion was particularly apparent in experiments wherein cultures were subjected to a selective pressure that favored failure of interference. For example, Jiang et al. (2013) introduced a conjugative plasmid encoding antibiotic resistance into *Staphylococcus epidermidis* RP62a, which had a type III-A CRISPR system and a spacer targeting the plasmid. When cultures were grown in the presence of antibiotics, interference against the plasmid resulted in 3–4 orders of magnitude fewer transconjugants as compared to controls. However, a few transconjugants were isolated and 13% of these had lost the plasmid-targeting spacer from their array. Other transconjugants had different mutations, all of which would disrupt CRISPR interference and thus allow the plasmid to persist and provide antibiotic resistance. Additional experiments suggested that these mutations arose spontaneously in the population rather than being induced by the selective pressure, and authors estimated that such mutations occurred in roughly one of 10^3^ or 10^4^ cells (Jiang et al., 2013).

In similar experiments with different organisms, spontaneous deletion of the targeting spacer was responsible for a larger share of escapees, in some cases occurring in more than 80% of the sequenced isolates (Gudbergsdottir et al., 2011; Lopez-Sanchez et al., 2012; Citorik et al., 2014; Rao et al., 2017; Stout et al., 2018; Canez et al., 2019). Deletion often included blocks of spacer-repeat units rather than only the targeting spacer (Gudbergsdottir et al., 2011; Lopez-Sanchez et al., 2012; Stout et al., 2018) and it was also sometimes associated with duplications of other non-targeting spacers (Lopez-Sanchez et al., 2012). As in the *S. epidermidis* work, rearrangements of the array were found even in the absence of selective pressure: for a strain of *Legionella pneumophila* bearing an engineered short array, roughly one of every 1,000–2,000 cells underwent a spontaneous spacer-repeat deletion (Rao et al., 2017). Sequencing revealed that the boundaries of the downstream repeat were maintained after the deletion, leading the authors to hypothesize that homologous recombination between repeats underlies array rearrangements (Figure 1B; Gudbergsdottir et al., 2011; Rao et al., 2017).

### Mechanisms and Functions for Polarized Spacer Uptake

Polarized, leader-end addition of spacers was a reproducible observation and mechanisms soon emerged to show how it occurs. Cas1 and Cas2 are necessary for spacer uptake (Yosef et al., 2012) and are associated with all adaptation-active systems (Makarova et al., 2020). These two proteins are necessary and sufficient for *in vitro* integration (Nunez et al., 2015). In some organisms, Cas1 and Cas2 strongly favor integration at the leader-adjacent repeat and this bias is mediated by sequences in the leader (Wei et al., 2015; McGinn and Marraffini, 2016; Wright and Doudna, 2016; Xiao et al., 2017; Kim et al., 2019). But for other systems, *in vitro* experiments show that Cas1 and Cas2 alone will carry out integrations at other repeats in the array and even at repeat-like sequences outside the array (Nunez et al., 2015; Grainy et al., 2019). These same systems show polarized integration *in vivo* (Datsenko et al., 2012; Yosef et al., 2012; Shiimori et al., 2018), suggesting that additional factors can guide the reaction. In type I systems of *E. coli* and other bacteria, a protein called integration host factor (IHF) ensures polarization by binding to Cas1 and the leader (Nunez et al., 2016; Fagerlund et al., 2017; Wright et al., 2017). Other protein factors likely play a similar polarizing role in other organisms and await characterization (Rollie et al., 2018).

Several studies show that leader-adjacent integration is likely necessary for optimal immune function. A new spacer arises from contemporary mobile genetic elements, which likely represent the most current and therefore pressing threats for host cells. In addition, new spacers should be free of mismatches that accumulate for older spacers as their targets develop escape mutations (Deveau et al., 2008; Semenova et al., 2011; Cady et al., 2012; Rao et al., 2016). From that we could expect that leader-adjacent spacers would be prioritized for defense, and these spacers do indeed produce more robust interference (McGinn and Marraffini, 2016; Rao et al., 2016; Deecker and Ensminger, 2020). The mechanism underlying this difference is not entirely clear. Leader-adjacent spacers (and the crRNA’s they encode) may be better expressed or more efficiently processed than downstream spacers. RNA sequencing data show more abundant crRNAs in the leader half of the array for many CRISPR loci (Elmore et al., 2013; Carte et al., 2014; McGinn and Marraffini, 2016). Another possibility rests on the idea that individual crRNAs are essentially in competition to form a crRNP effector complex with less numerous Cas nucleases. As the first to be transcribed, leader-adjacent spacers may have a head start in a race to associate with Cas proteins and could suffer the least from the “dilution” effect of multiple spacers (Martynov et al., 2017). Experiments with a constructed mini-array lent support to the general idea of competition: a truncated array was created in a strain of *Legionella pneumoniae* by deleting all but the leader-adjacent spacer and its upstream and downstream repeats. This mini-array strain showed about 100-fold more plasmid targeting than the wildtype strain, which has 42 additional spacers downstream (Rao et al., 2017). Though the sequence and position was identical for the first spacer, loss of additional spacers dramatically increased its effectiveness.

### CRISPR Arrays Vary in Length

In addition to influencing the polarity of spacer uptake, the dilution effect may also represent a functional constraint on the overall length of CRISPR arrays. Adaptation without spacer loss would presumably lead to ever-longer arrays, but among genomes sequenced so far, extremely long arrays are relatively rare. Arrays with greater than 100 spacers are observed; *Haliangium ochraceum* is a notable example, with a single array of 587 spacers and two other arrays measuring 189 and 36 spacers, respectively (Ivanova et al., 2010; Pourcel et al., 2020). However, a typical array contains fewer than 50 spacers in bacteria and fewer than 100 in archaea (Horvath et al., 2008; Mangericao et al., 2016; Pourcel et al., 2020). Array length does not appear to be limited by genome size (Pourcel et al., 2020) nor by cell resources: experimentally, lengthening an array by several spacers did not reduce fitness (Vale et al., 2015). Also, many genomes harbor more than one CRISPR system (up to 37 have been observed, in a species of *Actinoalloteichus*), and presumably the energy demands for a single 500 spacer array are similar to those for ten 50 spacer arrays. On the other hand, *cas* genes can have a fitness cost (Vale et al., 2015), so the observation that many organisms have evolved multiple short arrays suggests that array length is not limited by energetic costs of carrying extra spacers.

One hypothesis to explain array length patterns is that array size represents a tradeoff between the dilution effect described above and maintaining immunity to a range of potential threats, i.e., depth of immunity (Bradde et al., 2020). In turn, depth of immunity is balanced against the need to update the array frequently enough to contend with novel threats but not so frequently that the cell risks toxic auto-immunity (Stern et al., 2010; Vercoe et al., 2013; Weissman et al., 2018). Organisms may deal with dueling constraints by having multiple arrays, each with a different length and optimized depth of immunity (Weissman et al., 2018). This would imply that arrays can have different rates of both spacer uptake and loss. Regarding uptake, evidence already exists that adaptation efficiency varies among systems and can also change in response to certain cues like cell density (Hoyland-Kroghsbo et al., 2017; see Sternberg et al. (2016) for a general review of adaptation). Data on spacer loss is sparser, but at least one report suggests that the frequency of spacer loss can differ between systems in the same organism. Specifically, when otherwise identical plasmids with either a type I or type II mini-array were grown in *E. coli*, frequent spacer loss was observed for type I but not type II (Canez et al., 2019).

### Spacer Turnover Is Not Strictly Chronological

As a spacer’s residence time in an array increases, and it loses relevance, position, and sequence identity to its targets, we might expect selective pressures to no longer favor its maintenance. In that context, deletion events could be a useful means for shedding older spacers. However, multiple observations suggest that old spacers are not purged in a chronological manner and that mismatched or inefficient spacers may prove useful. A minority of older spacers can maintain identity to their protospacer targets, possibly due to stable or cyclical exposure to phages (Sun et al., 2016). We also now know that relatively ineffective spacers can participate in immunity through the process of primed adaptation. In short, priming occurs when a crRNP effector complex recognizes a protospacer target and then stimulates new spacer uptake using DNA located near that target (Datsenko et al., 2012; Swarts et al., 2012). Priming is observed even when interference is relatively inefficient, like when the protospacer does not have a canonical PAM or when there are mismatches between the crRNA and the protospacer, particularly in the “seed” region adjacent to the PAM (Semenova et al., 2011; Wiedenheft et al., 2011; Fineran et al., 2014; Li et al., 2014; Richter et al., 2014; Semenova et al., 2016; Garrett et al., 2020). Since primed adaptation tolerates these changes, a spacer that might otherwise be obsolete can contribute to CRISPR immunity by updating the CRISPR array. Experimentally, spacers in the middle of an array (*L. pneumophila*) were shown to give relatively inefficient interference but still effectively support priming (Deecker and Ensminger, 2020). Thus turnover of older spacers may not always appear steady or strictly chronological.

Many studies that demonstrated the polarity of spacer acquisition also described the relative stability of the array’s trailer end (Lopez-Sanchez et al., 2012; Weinberger et al., 2012; Lam and Ye, 2019). Assuming sequential spacer uptake, we would expect these terminal spacers to be the oldest and thus the most likely to have lost protective potential. Phylogenetic relationships inferred from multilocus sequence typing supported the idea that terminal spacers are indeed ancestral (Lopez-Sanchez et al., 2012). These spacers should be lost if shedding is chronological, yet they are apparently deleted far less frequently than newer spacers toward the middle of the array. Therefore trailer end spacers may be maintained for reasons unrelated to their value in interference. The stability may be a simple outcome of fewer opportunities for recombination: an internal spacer can be lost through recombination involving any upstream or downstream repeat, but the last spacer would only be lost if recombination occurred at the terminal repeat. Terminal repeats may also be stabilized due to polymorphisms: in many systems the repeat sequences are identical throughout the array except at the end (Jansen et al., 2002; Bolotin et al., 2005; Pourcel et al., 2005; Horvath et al., 2008; Lopez-Sanchez et al., 2012; Deecker and Ensminger, 2020; Refregier et al., 2020). Specifically, nucleotide substitutions or deletions are often found in the 3′ end of the terminal repeat (Figure 1C). Since identical repeats are most amenable to homologous recombination (Treangen et al., 2009), a trailer repeat without polymorphisms could potentially undergo recombination with the leader-adjacent repeat and eliminate the entire array, leaving only a copy of itself. Terminal repeat polymorphisms may thereby tend to reduce the likelihood of array collapse.

What would array collapse mean for immunity in the CRISPR locus? Experiments have confirmed that naïve adaptation can occur with a solitary leader-adjacent repeat (Yosef et al., 2012; Wei et al., 2015), which suggests that arrays could potentially be repopulated following a collapse, at least in laboratory conditions. Deecker and Ensminger (2020) also found evidence that “array collapse and repopulate” events occur naturally. First they showed that priming *in trans* could replenish a collapsed array in the lab: their strain of *L. pneumophila* naturally contains a type I-F system on both its chromosome and its endogenous plasmid. The chromosomal array was mutated to only contain the terminal repeat, mimicking a collapsed array. They transformed in a plasmid targeted by a spacer in the endogenous plasmid array and observed primed adaptation into the chromosomal collapsed array. The authors noted that patterns of repeat polymorphisms among naturally occurring *L. pneumophila* isolates looked like the replenished arrays they had created in the lab, suggesting this happens in nature. However, it remains unclear whether replenishment of a collapsed array is a universal phenomenon. If collapse is not well tolerated, terminal repeat polymorphisms may be functionally important in preventing it. On the other hand, if a system can readily bounce back from array collapse, terminal repeat polymorphisms may simply represent spontaneous mutations that persist because they are resistant to loss through recombination. Interestingly, many terminal repeat polymorphisms are nucleotide substitutions or truncations in the 3′ end, which partially disrupt the loose dyad symmetry of repeat ends (Jansen et al., 2002; Bolotin et al., 2005; Pourcel et al., 2005; Horvath et al., 2008; Lopez-Sanchez et al., 2012; Deecker and Ensminger, 2020). Since dyad symmetry is a frequently observed feature of repeats, one could speculate that loss of dyad symmetry helps stabilize terminal repeats.

From the studies discussed above, we can conclude that the trailer end of the array typically does not obey a pattern of chronological turnover. In an extreme example, spacer turnover across the entire array also bucks chronological turnover, even over the course of thousands of years. Savitskaya et al. (2017) acquired an intestinal microbiome sample from a well-preserved mammoth calf that was frozen for 42,000 years and they captured *E. coli* type I repeat-spacer amplicons by PCR. Reads primarily yielded data about individual spacers but a subset of reads were long enough to span two or three repeat-spacer units and these provided additional information about spacer order in the ancient arrays. Ancient spacers and spacer combinations were then compared to over 1,700 modern *E. coli* type I-E arrays from public databases. About 20% of the ancient spacers matched a modern spacer, and surprisingly those matches were positioned all over the modern arrays rather than being concentrated in the distal end. Trends for the two and three-spacer data were similar. This striking example demonstrated that for some systems, spacer order may not recapitulate a timeline of spacer acquisition.

### Mechanisms for Spacer-Repeat Rearrangement

Repeats have often been associated with genome plasticity, and rearrangement of a repeat element like the CRISPR array is consistent with those observations. Repeats can undergo recombination through two general mechanisms: RecA-dependent homologous recombination and RecA-independent mechanisms like replication misalignment (slippage or slipped-strand mispairing) (Bzymek and Lovett, 2001; Treangen et al., 2009). In homologous recombination, RecA protein plays a key role as it binds and coats ssDNA and promotes strand exchange and annealing once it has found a region with sufficient sequence identity. The branched heteroduplex is then extended and resolved by, for example, RuvABC complex (Kowalczykowski, 2015). RecA-independent mechanisms also rely on homology but there are multiple distinguishing characteristics. First, RecA-independent mechanisms have shorter homology length requirements and are thought to be the primary source of recombination in prokaryotes for repeats that are less than about 200 bp (Bi and Liu, 1994; Lovett, 2004). Second, RecA-independent recombination is thought to involve the replication fork: in replication misalignment (the most well-described form of RepA-independent recombination), direct repeats mispair during replication, giving rise to duplications and deletions (Lovett, 2004). The frequency of this type of recombination increases with repeat length and identity and decreases as the spacing between repeats grows (Lovett, 2004). Disruption of replication can further promote misalignments and increase deletions (Michel, 2000).

**FIGURE 2:**
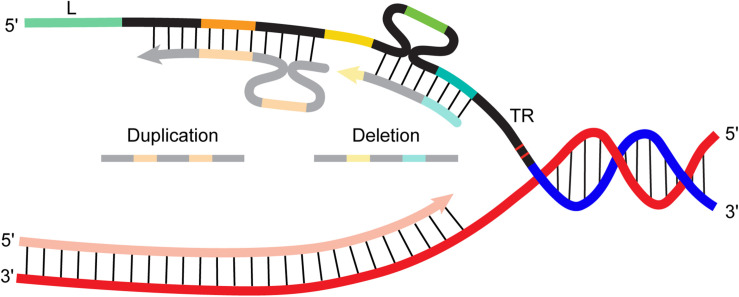
Cartoon diagram of spacer-repeat duplication and deletion events created by misalignment of the nascent strand during replication. L, leader; TR, terminal repeat, all other repeats depicted in black, spacers depicted in color (*orange*, *yellow*, *green*, *aqua*).

Given that CRISPR repeats are short and closely spaced, a RecA-independent mechanism like replication misalignment could underlie array deletions and duplications. RecA is not necessary for adaptation (Ivancic-Bace et al., 2015; Radovcic et al., 2018). While there is not yet direct evidence for or against a role for RecA in CRISPR array rearrangements, a report about recombination in a CRISPR-derived system hints that it is not necessary. Ding et al. (2020) sought to improve the performance of dual guide RNA plasmids in CRISPR-based genome editing applications. The plasmids were designed to express two separate guide RNAs: each 20 bp guide spacer had an identical promoter (35 bp) upstream and identical “scaffold” (82 bp) downstream (the scaffold included 12 bp corresponding to the 5′ end of the CRISPR repeat, a 4 bp linker, and “tracr”, the trans-activating crispr RNA important for forming a mature guide RNA). They found that the plasmid was extremely unstable: 73% had mutations, mostly deletions that excised either one of the two promoter-spacer-scaffold units. Changing the promoter to reduce the extent of homology did not eliminate deletions. The group also observed no reduction in deletion frequency using strains with deleted or inactive RecA, showing that the process was not RecA dependent. Ultimately the group found that inverting one of the promoter-spacer-scaffold units was necessary to stop the deletion events. Interestingly, the authors found that growth and transformation conditions also influenced the frequency of deletions. Using electroporation instead of heat shock and culturing in rich growth medium both reduced (but not eliminated) deletions. They hypothesized that nutrient deprivation and DNA damage slow replication and thereby promote deletion through a replication mispairing mechanism on the lagging strand. These findings might not be directly applicable to native CRISPR arrays (for example, the homologous region in their engineered plasmid was longer—82 bp when different promoters were used), but they imply that natural spacer deletions could also be RecA-independent, possibly occurring through misalignment between repeats during replication (Figure 2).

## Discussion

Bringing together these different observations and experimental results, we can speculate on a general model for spacer dynamics in a CRISPR array: new spacers are added at the leader end of the array at some basic frequency, which varies among species, systems, and conditions. Rearrangement of the array is ongoing at some level, though the particular frequency is also variable among species and CRISRR-Cas classes and it may be modulated by as-of-yet unidentified factors and conditions. These rearrangements can lead to both deletions and duplications, and the interplay between spacer addition and loss determines array length and underpins the balance of immune depth, immune novelty, and crRNA dilution for that array. The terminal spacer-repeat unit rarely participates in rearrangements, potentially because of polymorphisms, so the array is maintained and the last spacer-repeat unit is stable. Together with adaptation events, rearrangements present immunogenic diversity on which selection can act. In most circumstances the dominant array form persists for generations, but the system is poised for change should conditions shift.

There is much more to learn about the dynamics and outcomes of spacer turnover. For one, it will be interesting to know how common array rearrangements are in different natural populations. Often these events were only detected because of a strong selective pressure against interference—how frequent are they in a native array under neutral conditions? Is the frequency consistent or does it vary with or independently from adaptation frequency? Since evidence suggests that the arrangements may not be equally common for all systems (Canez et al., 2019), it will be worthwhile to explore their frequency in multiple species and conditions. Long-read sequencing approaches may be particularly suitable for these experiments since they can capture the spacer composition of an entire array without the ambiguities inherent to assembled short reads.

Second, we have much to learn about the mechanism by which spacers are duplicated or deleted. The nature of CRISPR repeats and patterns of spacer loss are suggestive of rearrangement by recombination, but direct data are needed. For example, do deletions and duplications arise from RecA-independent mechanisms like misalignment in the replication fork? This would be supported by the results from the dual guide RNA plasmid experiments described above (Ding et al., 2020), and if experimentally confirmed, it could have interesting implications for immune diversity. If, for example, deletions primarily occur on the lagging strand during replication, we would expect them to be passed along to only one of two daughter cells. Since autoimmunity is thought to represent a fitness cost associated with CRISPR-Cas (Stern et al., 2010; Vercoe et al., 2013), replication fork deletion of new spacers may present a way to hedge against toxic self-targeting adaptation. If replication fork misalignment does underlie array rearrangements, are there factors or conditions that promote or inhibit the process and do they regulate spacer maintenance? And looking beyond the replication misalignment model, are there other enzymes or processes that can lead to spacer deletions or duplications? As we have learned more about adaptation and interference, points where the processes are modulated have been uncovered. Similarly, answering these and other questions about array dynamics may also help us uncover novel mechanisms that govern how existing spacers are managed to optimize immunity.

## Author Contributions

SG devised and wrote the manuscript.

## Conflict of Interest

The author declares that the research was conducted in the absence of any commercial or financial relationships that could be construed as a potential conflict of interest.
